# Modification of Alkali Lignin with Poly(Ethylene Glycol) Diglycidyl Ether to Be Used as a Thickener in Bio-Lubricant Formulations

**DOI:** 10.3390/polym10060670

**Published:** 2018-06-16

**Authors:** Esperanza Cortés-Triviño, Concepción Valencia, Miguel A. Delgado, José M. Franco

**Affiliations:** Pro2TecS-Chemical Product and Process Technology Centre, University of Huelva, 21071 Huelva, Spain; esperanza.cortes@diq.uhu.es (E.C.-T.); barragan@uhu.es (C.V.); miguel.delgado@diq.uhu.es (M.A.D.)

**Keywords:** epoxide-functionalized lignin, castor oil, lubricating greases, rheology, tribology

## Abstract

Considerable efforts are currently being made by the academic community and industry, aiming to develop environmentally friendly lubricants with suitable technical features for their performance. In this context, lignin could be considered a promising candidate to be used as a bio-sourced thickening agent to formulate eco-friendly lubricating greases. In this work, alkali lignin (AL) was chemically modified with poly(ethylene glycol) diglycidyl ether (PEGDE). Afterwards, the epoxidized lignin was properly dispersed in castor oil (CO) in order to obtain an oleogel for lubricant applications. The epoxidized lignins were characterized by means of epoxy index determination, thermogravimetric analysis (TGA) and Fourier transform infrared (FTIR) spectroscopy. The epoxide-functionalized lignin-based oleogels were analyzed from both rheological and tribological points of view. It was found that the viscosity, consistency and viscoelastic functions of these oleogels clearly increased with the epoxy index of the epoxide-modified lignin compound. Thermo-rheological characterization of these oleogels revealed a slight thermal dependence of the viscoelastic moduli below 100 °C, but a significant softening above that critical temperature. In general, these oleogels showed low values of the friction coefficient under the mixed lubrication regime as compared to the neat castor oil.

## 1. Introduction

Several million tonnes per year of used lubricants in Europe are ultimately poured into the environment as a result of leaks, spillages or other problems [1]. Consequently, this worldwide environmental concern has led to a growing interest in the use of renewable and environmentally friendly materials in lubricant formulations. Moreover, motivations to design new biodegradable materials are also induced by government incentives, public opinion, corporate programs of social and environmental responsibility, and response to marketing needs.

In the particular case of lubricating greases, the most feasible measure to be implemented by manufacturers in order to produce environmentally friendly formulations is the replacement of the mineral or synthetic lubricating oil, which comprises 70–90 wt % of grease components, as for instance established in the Blue Angel eco-label in 1978 [2]. For this purpose, it is well known that vegetable oils are gaining more importance in environmentally friendly industrial applications due to their inherent biodegradability, renewability, low ecotoxicity and low toxicity towards humans [3,4]. In particular, castor oil seems to be a good option due to the presence of hydroxyl groups in its chemical structure, which provide exceptional characteristics of lubricity [5,6,7].

However, the most challenging issue is the substitution of traditional thickening agents, such as lithium, aluminium, sodium and calcium soaps or polyureas, with other types of more environmentally friendly materials, in order to produce fully biodegradable lubricating greases. In this sense, Sánchez et al. [8] and Núñez et al. [9] evaluated the performance of cellulose derivatives-based oleogels and gel-like dispersions of industrial cellulose pulps, respectively. The potential use of chitin, chitosan and some acylated derivatives as biogenic thickening agents to formulate stable dispersions in vegetable oils was explored by Sánchez et al. [10]; whereas Gallego et al. [11] and Borrero-López et al. [12] reported the preparation of oleogels using NCO-functionalized lignocellulosic materials for the same use.

Lignin is one of the most abundant natural polymers present in plant biomass, and its chemical structure is characterized by phenylpropanoid units, such as *p*-hydroxyphenyl, guaiacyl and syringyl moieties, forming a complex three-dimensional network which comprises numerous inter-unit bonds, including ether and carbon-carbon linkages [13,14,15,16]. Lignin, possessing a complex and non-well defined structure, is generally considered a waste material due to its limited industrial reutilization up to now [16]. However, the existence of numerous functional groups susceptible to modification in its structure, like aliphatic and phenolic hydroxyl groups, makes possible different potential uses of lignin in several applications [17,18]. In this way, the epoxidation reaction of lignin through hydroxyl groups functionalization, among other chemical modifications, allows one to increase their chemical reactivity intensifying the lignin potential and its valorization [13,14,17,18,19,20].

In previous work, lignin was epoxidized using epichlorohydrin, and then was dispersed in castor oil thus resulting in gel-like systems stabilized by chemical crosslinking [21]. However, the toxicity of epichlorohydrin and the limited rheological properties imparted to the gel-like dispersion require another pathway for lignin epoxidation to be explored. Bearing in mind these assumptions, this work focused on finding a distinctive alternative for lignin valorization by using it as an effective thickening agent in castor oil-based lubricants through the study of the epoxidization of an alkali lignin with poly(ethylene glycol) diglycidyl ether (PEGDE). The influence of lignin/PEGDE ratio used in the epoxidation reaction and concentration of epoxide-functionalized lignin on the performance properties of the resulting oleogels, i.e., rheological, tribological and thermal characteristics, were studied.

## 2. Materials and Methods

### 2.1. Materials

Castor oil was supplied by Guinama (Valencia, Spain). Composition and main physical properties can be found elsewhere [22]. Poly(ethylene glycol) diglycidyl ether (PEGDE), with an average molecular weight (*M*_n_) of 500 g/mol was used to chemically modify a commercial alkali softwood lignin (AL) obtained from a Kraft process. Both of them were supplied from Sigma-Aldrich (St. Louis, MO, USA).

### 2.2. Preparation of Epoxide-Functionalized Alkali Lignin (EAL)

In a typical facile procedure, 10 g of alkali lignin and 150 g of a 20 wt % sodium hydroxide solution were placed into a round-bottom flask mounted with a thermometer, a magnetic stir bar and dropping funnel and then stirred at 30 °C until dissolution. Then, different amounts of PEGDE (see Table 1) were added dropwise and the reaction was conducted for 3 h. Finally, the mixture was centrifuged at 4000 rpm, filtered and dried in an oven at 80 °C.

### 2.3. Preparation of Oleogels

EAL samples were dispersed in castor oil by mixing at 70 rpm with a RW-20 device (Ika, Staufen, Germany) using an anchor impeller. EAL concentration was varied between 2.5 and 10 wt %. This concentration range was selected based on physical stability and suitability of mechanical properties. The mixture was kept under agitation at room temperature for 24 h and then homogenized with an Ultra-Turrax T25 (Ika, Staufen, Germany) rotor-stator turbine, at 10,000 rpm. A reference system comprised of an unmodified lignin/sodium hydroxide blend and castor oil was also prepared in order to quantify the epoxide contribution on the oleogels’ performance. 

### 2.4. Epoxy Index Determination

The presence of epoxy groups in the modified lignin was determined by titration of a mixture of the epoxidized compound with chloroform, glacial acetic acid, tetraethylammonium bromide and crystal violet with perchloric acid according to ISO 3001:1999 (E).

### 2.5. NaOH Residual Analysis

The samples were washed several times with distilled water in order to quantify the amount of residual NaOH in the epoxidized lignins. The collected washing water was titrated with hydrochloric acid solution (0.1 N).

### 2.6. Fourier Transform Infrared (FTIR) Spectroscopy

FT/IR-4200 spectrometer (JASCO, Tokyo, Japan) was used to obtain FTIR spectra within the wavenumber range from 4000 to 400 cm^−1^, at 4 cm^−1^ resolution, in the transmission mode. PEGDE-functionalized lignins were prepared as KBr pellets and placed into a holder.

### 2.7. Thermal Analysis (TG/DTA)

Thermogravimetric analysis was carried out by using an Exstar TG/DTA 6200 (Seiko, Chiba, Japan) under purging with nitrogen. 10–15 mg of each sample was placed on a platinum pan, and heated at 10 °C/min from 30 to 600 °C.

### 2.8. Rheological Characterization

The rheological characterization of oleogels was performed in a Physica MCR 501 (Anton Paar, Graz, Austria) rheometer, at 25 °C, using a plate-plate geometry (25 mm diameter, 1 mm gap). Small-amplitude oscillatory shear (SAOS) tests were carried out in a frequency range of 0.03 and 100 rad/s, within the linear viscoelasticity regime. The linear viscoelasticity range was previously determined by performing stress sweep tests at 1 Hz. Samples were measured after 24 h and 2 months of storage time. Moreover, SAOS tests were also performed at different temperatures (between 5 and 150 °C) for selected samples.

In addition, viscous flow tests were carried out 2 months after preparation. The viscosity of these oleogels was measured in a shear rate range of 10^−2^–10^2^ s^−1^, at 25 °C, using a rough surface plate-plate geometry (25 mm diameter, 1 mm gap) in order to avoid wall-slip phenomena [23]. At least two replicates of each test were done on fresh samples.

### 2.9. Penetration Tests

A Seta Universal penetrometer, model 17000-2, with one-quarter cone geometry (Stanhope-Seta, Chertsey, UK) was used to measure the penetration indexes of epoxidized lignin-based oleogels according to the ASTM D 1403 standard. The penetration values were converted to full-scale penetrations following the ASTM D 217 standard.

### 2.10. Tribological Measurements

Friction tests were performed in a ball-on-three-plates tribological cell [24] coupled to a Physica MCR 501 rheometer (Anton Paar, Graz, Austria). The tribological cell consists of a lower measuring geometry designed as a reservoir for the lubricant, with three 45° inclined steel plates (1.4301), while the upper measuring geometry keeps fixed a 6.35 mm 1.4401 grade 100 bearing ball. The ball was fixed to avoid rolling and slides on the three plates. This ball-on-three-plates configuration makes it possible to determine the friction coefficient, defined as the ratio between the applied normal force and the friction force measured by the rotational rheometer. The friction coefficient was monitored by applying a rotational speed sweep (0–1000 rpm) and 10 N of normal force, at room temperature (~25 °C). At least six replicates of each test were done on fresh sample.

## 3. Results and Discussion

### 3.1. Lignin Epoxidation with PEGDE

Epoxidation of a variety of lignins with epichlorohydrin has been previously investigated [21,25,26,27]. In these studies, the effects of temperature, reaction time, NaOH/lignin ratio and epoxy/lignin ratio on the chemical modification of lignin were analyzed. Cortés-Triviño et al. [21] and Malutan et al. [25] pointed out that high reaction temperatures had a negative effect on epoxidation due to the occurrence of secondary reactions. Thus, a relatively low temperature (30 °C) was selected in this research. The epoxy index decreased with reaction time, probably due to crosslinking reactions, although this effect may depend on both epoxy/lignin ratio and temperature [25]. In this sense, Ferdosian et al. [26] and Lai et al. [27] proposed the optimum reaction time range between 2.5 and 5 h. NaOH/lignin ratio tends to increase the gross weight of alkali lignin-PEGDE compound and affects the solubility of lignin. In this work, the alkali load was 20 wt % to obtain a pH value around 12–13, which is necessary for a complete dissolution of the alkali lignin powder. Consequently, a chemical lignin structure in the form of a sodium salt should be obtained, which could be of interest for the intended lubricant application, due to the use of sodium soap as thickener in the formulation of lubricating greases [28]. Therefore, taking into account that lignin/epoxy ratio is the factor exerting the most significant effect on the epoxide-modified lignin [26], several samples differing in the input PEGDE/AL weight ratio (between 0.25 and 5) were prepared by maintaining the reaction temperature at 30 °C, the reaction time at 3 h and the alkali load at 20 wt %. (see Table 1). In all cases, AL was properly epoxidized, resulting in a dark-brown powder after three hours of reaction.

Table 1 shows the epoxy index values as a function of the PEGDE/AL ratio, as well as the residual amount of NaOH in the EAL powder. Significant differences in both the epoxy index and the amount of NaOH were obtained by modifying the PEGDE/AL ratio. Thus, the lower the PEGDE/AL ratio, the higher the epoxy index and the residual sodium hydroxide. As was previously pointed out by Delmas et al. [28], two competitive reactions occur during the intermediate step of epoxidation at high pH; on the one hand, a partial etherification of lignin with the epoxy group of PEGDE and, on the other hand, the alkaline hydrolysis of PEGDE into hexahydroxy-PEG. In relation to the alkaline hydrolysis of PEGDE, it is worth pointing out that the PEGDE oxirane ring opening leads to an intermediate sodium alkoxide group that can either be converted into a hydroxyl group or attack the oxirane group of another PEGDE molecule resulting in a chain-growth polymerization [28]. Consequently, high PEGDE/AL ratios favor the alkaline hydrolysis of PEGDE, which decreases the residual amount of sodium hydroxide and prevent the epoxidation of the hydroxyl groups in the lignin to obtain the epoxide-modified lignin compound, thus resulting in lower values of the epoxy index. Therefore, the highest epoxy index was achieved in sample EAL1, which was prepared with the lowest PEGDE/AL ratio (0.25/1).

**Figure 1:**
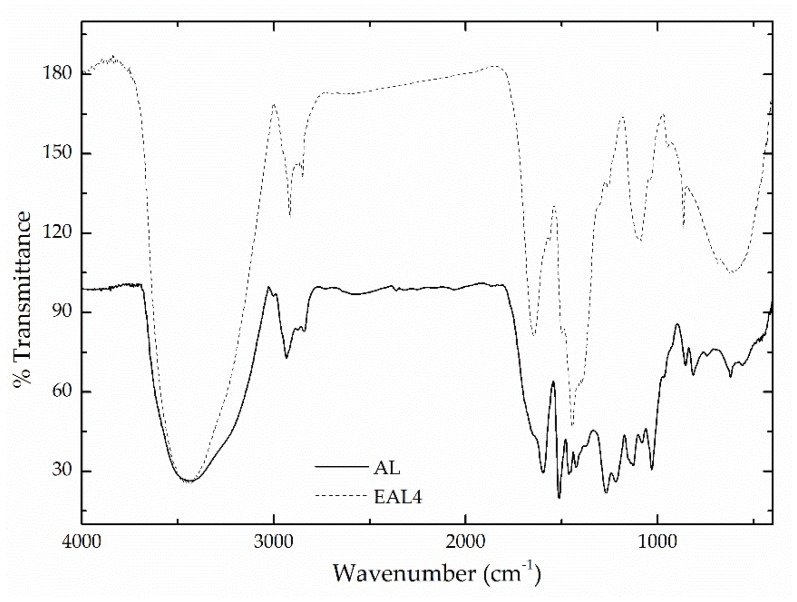
FTIR spectra for the original alkali lignin and a selected epoxide-modified lignin (EAL4).

FTIR spectroscopy was employed to verify the structural changes resulted after lignin epoxidation. Figure 1 shows the spectra for both AL and a selected epoxidized sample (EAL4). As was previously reported [29], AL shows a variety of intense peaks, which can be attributed to the O–H stretching vibration (3440 cm^−1^), asymmetric (2917 cm^−1^) and symmetric (2850 cm^−1^) stretching vibration of methylene, methyl and methoxyl groups, aromatic ring vibrations of the phenyl propane skeleton (1600, 1510 and 1426 cm^−1^), C–H deformations of asymmetric methyl and methylene groups (1459 cm^−1^), C–O stretching vibration of guaiacyl units (1272 cm^−1^), C–H of guaiacyl and syringyl (1130 cm^−1^), and vibrations of C–O in secondary (1080 cm^−1^) and primary alcohol groups (1030 cm^−1^). However, significant differences with EAL4 spectrum can be observed within the wavenumber range of 500–2000 cm^−1^. It is well known that the presence of NaOH significantly masks the IR absorption peaks of a given sample, especially in the range of 1300–1500 cm^−1^ [30]. Despite this fact, the epoxide modification of the alkaline lignin can be verified through the appearance of a characteristic absorption peak of the epoxy group (oxirane ring vibration) at 865 cm^−1^. In addition, an absorption peak at 1126 cm^−1^ assigned to the stretching vibration of C–O–C in the PEGDE structure can be also observed [31,32].

**Figure 2:**
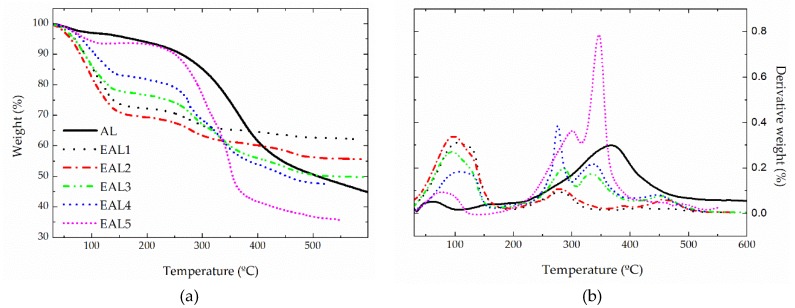
Loss weight (**a**) and decomposition-rates (**b**)curves during the thermal degradation for the original alkali lignin and epoxidized lignin samples.

TGA analysis was also applied to study the thermal decomposition of epoxidized lignins aiming to elucidate the effectiveness of functionalization. Figure 2 shows the thermogravimetric curves, in the form of both weight loss and rate of weight loss (first derivative), of the original alkali lignin (AL) and the epoxide-modified lignin samples (EALs) studied. More relevant thermal degradation data calculated from the corresponding thermogravimetric curves are collected in Table 2.

As previously described [21,26], apart from the initial weight loss of around 5% at 50–100 °C due to moisture, the thermal degradation of this alkali lignin comprises the dehydration of hydroxyl groups at around 150 °C and the cleavage of ether linkages at around 275 °C. At this temperature, volatile gases such as CO, CO_2_, and CH_4_ [15,33] are produced, with subsequent pyrolytic degradation, which involves the cleavage of carbon-carbon linkages at a *T*_max_ around 380 °C. In consequence, the complete rearrangement of the backbone at the end of the test leads to 47% char, in agreement with previous investigations [15,34].

The residual amount of NaOH also influences the thermal decomposition of epoxidized lignin samples [35]. The great weight loss displayed in all samples between 80 and 150 °C is basically the consequence of the drying process performance, which does not completely remove the moisture from the solid phase after lignin epoxidation. However, the dehydration of newly generated hydroxyl groups as a consequence of the epoxidation process may also occur at these temperatures [36]. As a consequence, higher mass losses at around 150 °C were detected for more epoxidized samples. Apart from these dehydration processes, in general, the thermal degradation of epoxide-modified lignin samples started at temperatures similar to those of alkali lignin (200–250 °C). However, additional thermal events due to both the degradation of non-lignin components (PEGDE and hexahydroxy-PEG) and the cleavage of aliphatic hydroxyl groups [26,28] are apparent in the form of sharp peaks in the derivative curve at around 270–300 °C. A weight loss of within 6–25 wt % was observed in this temperature range, increasing with the PEGDE/AL ratio. These results are in agreement with those reported by Kleen and Gellerstedt [37], who stated that the presence of Na^+^ cations leads to a significant decrease in volatile pyrolysis products and facilitates the cleavage of hydroxyl and methoxyl groups. In addition to this, it was found that EAL1 and EAL2 showed the highest amount of residue, 62 and 57 wt %, respectively; while only 34 wt % char formation was achieved for EAL5. These results are also in accordance with other studies [35], which pointed out that char yield was inversely proportional to the total amount of hydroxyl and methoxyl groups in the lignin. As previously discussed, higher PEGDE/AL ratios inhibit the etherification of the hydroxyl groups in the lignin.

Alternatively, the main peak related with the pyrolytic degradation of epoxide-modified lignin was also highly affected by the PEGDE/AL ratio. In this way, changes in the distribution of functional groups and the cross-linking induced by epoxy groups may influence the carbon-carbon cleavage among lignin structural units [15,34]. Thus, the fragmentation of inter-unit linkages was shifted to temperatures higher than 400 °C for EAL1 and EAL2 samples, whereas much lower temperatures (325–350 °C) were found for EAL3, EAL4 and EAL5 samples, corroborating the lower epoxidation degree reached in these samples.

### 3.2. Rheological Characterization of Oleogels

**Figure 3:**
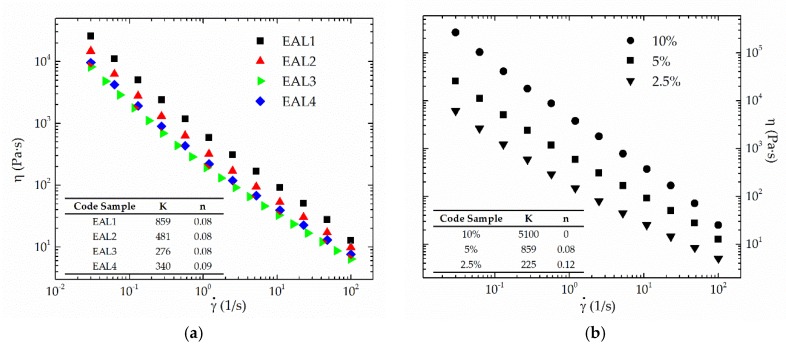
Viscous flow curves at 25 °C for epoxidized lignin-based oleogels as a function of (**a**) lignin epoxy index (5 wt % EAL) and (**b**) lignin concentration (EAL1 sample).

The dispersion of epoxidized lignin samples in castor oil produces physically stable oleogels, except for the case of the EAL5 sample with the lower epoxy index, which shows phase separation immediately after preparation. This suggests the occurrence of chemical gelation via chemical interactions between castor oil hydroxyl groups and the remaining epoxy groups, as previously proposed for oleogels prepared with epichlorohydrin-functionalized lignins [21]. Figure 3 shows viscous flow curves of stable epoxide-functionalized lignin-based oleogels, as a function of both epoxy index (Figure 3a) and thickener concentration (Figure 3b). A power-law model (Equation (1)) satisfactorily describes the shear-thinning flow behavior observed within the shear rate range analyzed (*r*^2^ > 0.995):

(1) $$\eta =k{\dot{\gamma }}^{n-1}$$

where η is the viscosity, $\dot{\gamma }$ the shear rate and “*k*” and “*n*” are consistency and flow indexes, respectively. The values of “*k*” and “*n*” parameters are displayed in the insets in Figure 3a,b. As can be seen, extremely low values of the flow index were obtained for all samples, characteristic of the typical yielding flow shown by traditional lubricating greases [38]. The consistency index clearly increases with both the epoxy index and thickener concentration, which is in agreement with penetration values shown in Table 3. These penetration values serve to classify lubricating greases in terms of NLGI grades [39], which represent a measure of grease consistency or hardness (see Table 3). In addition, the lowest penetration value was obtained for the oleogel prepared with EAL1 sample at 10 wt % concentration. Henceforth, as can be expected, a lower PEGDE/AL ratio, i.e., higher epoxy index, favors the chemical interaction between epoxy and the hydroxyl group of ricinoleic fatty acid chain.

**Figure 4:**
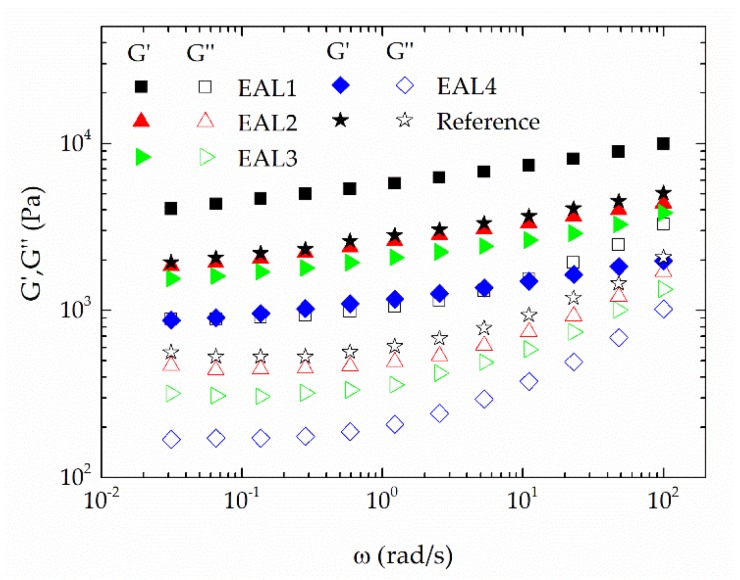
Evolution of the storage (*G*′) and loss (*G*″) moduli with frequency for epoxidized lignin-based oleogel as a function of lignin epoxy index (5 wt % EAL). Star symbols correspond to the reference system prepared with non-epoxidized lignin and NaOH in the same proportion than sample EAL1. (*G*′, filled symbols; *G*″, empty symbols).

Figure 4 shows the evolution of viscoelastic functions with frequency for the oleogels formulated with 5 wt % of several epoxidized lignin samples. In all cases, rheological measurements were done 24 h after oleogel preparation. These oleogels were compared with a reference prepared with non-epoxidized lignin and NaOH in the same proportion as in the oleogel containing the EAL1 sample. This reference was introduced in order to differentiate the effect of the residual NaOH in epoxidized samples on the rheological response of oleogels from the effect of lignin epoxidation.

As can be seen in Figure 4, the linear viscoelasticity response supports the idea that highly structured gels were obtained [38,40], where the values of the storage modulus, G′, were always higher than those of the loss modulus, G″, throughout the whole frequency range studied. The values of these viscoelastic moduli increased with the decrease of PEGDE/AL ratio, i.e., with the increase of the epoxy index, as a consequence of the higher density of crosslinking points induced by the reaction of epoxy and hydroxyl groups. On the other hand, the high amount of NaOH present in the highly epoxidized lignin sample brings about an additional stabilizing effect due to partial saponification of castor oil, as can be inferred from the rheological response of the reference. However, the effect of the chemical interaction between castor oil and the epoxidized lignin is well illustrated in Figure 4, where it is apparent that the oleogel prepared with the EAL1 sample showed values of both the storage and loss moduli significantly higher than the reference.

**Figure 5:**
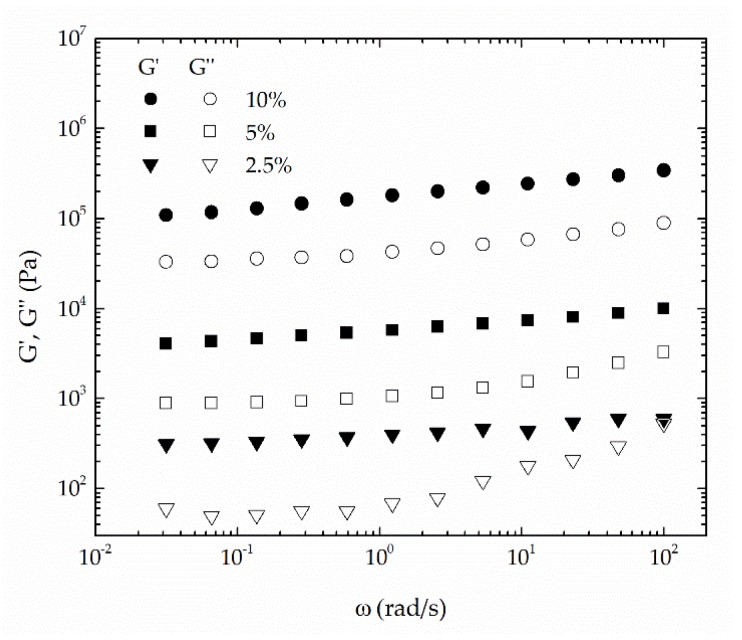
Evolution of the storage (*G*′) and loss (*G*″) moduli with frequency for epoxidized lignin-based oleogels prepared with sample EAL1 at different concentrations (*G*′, filled symbols; *G*″, empty symbols).

In addition, the influence of thickener concentration on the rheological behavior of these oleogels was studied for the most epoxidized lignin sample (EAL1). Figure 5 shows that the values of both storage and loss moduli increase with thickener concentration, which is also in accordance with the consistency index (Figure 3a) and penetration values (Table 3). It is worth emphasizing that the frequency dependence of SAOS functions is affected by the epoxide-functionalized lignin content. As can be seen, the minimum in the loss modulus, found at intermediate frequencies, was shifted to lower frequencies by decreasing EAL1 concentration. Moreover, a crossover between both SAOS moduli was detected at high frequencies for the 2.5 wt % concentration.

**Figure 6:**
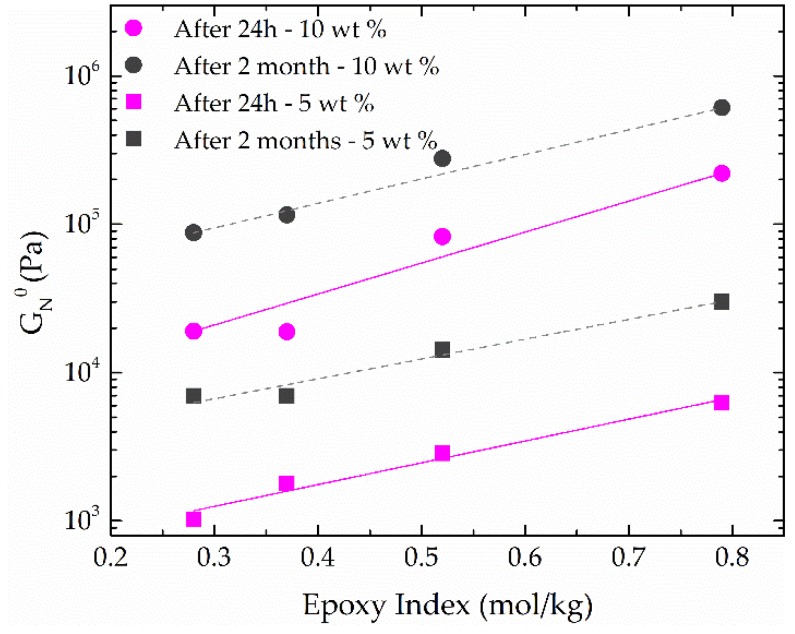
Evolution of *G*_N_^0^ with lignin epoxy index for EAL-based oleogels (5 and 10 wt % EAL) 24 h and 2 months after preparation.

In order to obtain more information about the influence of functionalization degree on the rheological behavior of these epoxide-functionalized lignin-based oleogels, the plateau modulus, *G*_N_^0^, was estimated as shown elsewhere [41]. *G*_N_^0^ values for oleogels containing 5 and 10 wt % of modified lignin, as a function of epoxy index, are shown in Figure 6. *G*_N_^0^ was calculated from SAOS measurements one day and two months after oleogels preparation, resulting in quite different values as a consequence of the progress of crosslinking reaction. It is worth mentioning that, as expected, the reference system, i.e., the NaOH/non-modified lignin-based oleogel, did not show any change in the rheological response along time because of the lack of active groups. Moreover, the increase of plateau modulus with the rise of epoxy index was apparent for both thickener concentrations, following the same trend, i.e., approximately the same power-law exponent, which indicates an increase of inter-unit linkages. In addition to this, an internal curing process takes place during the 2 months after oleogel preparation, resulting in a significant increase of SAOS functions with aging time (Figure 6).

**Figure 7:**
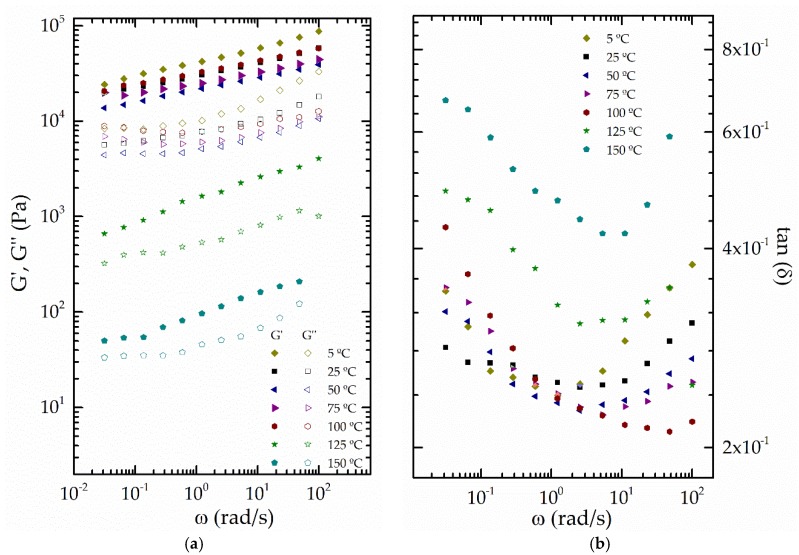
Frequency dependence of the storage and loss moduli (**a**) and the loss tangent (**b**) for a selected epoxidized lignin-based oleogel (EAL1 at 5 wt %) as a function of temperature (*G*′, filled symbols; *G*″, empty symbols).

**Figure 8:**
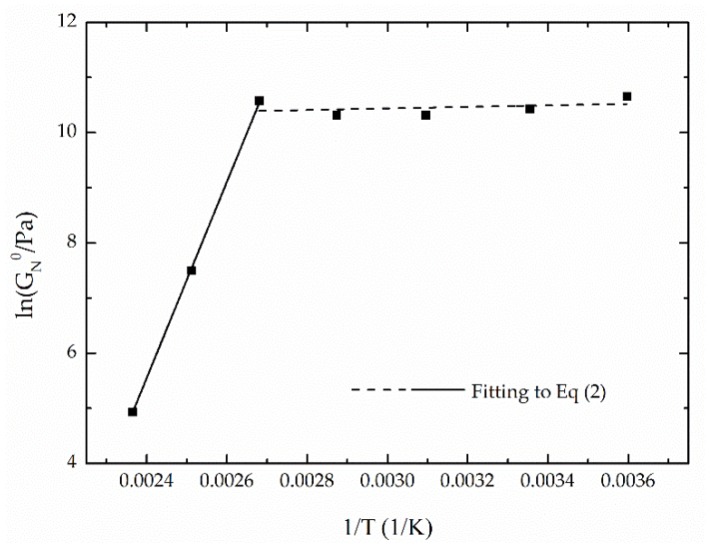
Evolution of *G*_N_^0^ with temperature for a selected oleogel (EAL1 at 5 wt %).

As is well known, it is also important to evaluate the effects of temperature on the rheological behavior of semi-solid lubricants [42]. In this sense, Figure 7 shows the evolution of viscoelastic moduli with frequency for the selected oleogel over a wide range of temperature. As can be observed, the values of *G*′ and *G*″ tend to decrease with the increase of temperature more dramatically above 100 °C, which is also associated with a significant increase of the loss tangent values, i.e., lower relative elasticity (Figure 7b). The effect of temperature on the plateau modulus is analyzed in Figure 8. A noticeable change in the evolution of *G*_N_^0^ versus 1/T was found at around 100 °C, which indicates a significant softening above this temperature. This result resembles the thermo-rheological behavior reported by Delgado et al. [42] for traditional lithium greases. As previously proposed [11,43], this thermal dependence of the plateau modulus can be fitted to two different Arrhenius-type relationships in the ranges of 5 to 100 and 100 to 150 °C, respectively:

(2) $$G_{0}^{N}=A\cdot e^{\frac{E_{a}}{R}\cdot \frac{1}{T}}$$

where *Ea* (J/mol) evaluates the thermal dependence in each temperature range, *R* is the gas constant (8.314 J/mol K), T is the absolute temperature (*K*), and *A* is the pre-exponential factor (Pa). Table 4 shows the fitting parameters of this equation in both temperature ranges for all oleogels studied. In all cases, low *E*_a_ values were obtained within the low temperature range, which indicates a very low thermal dependence of *G*_N_^0^. However, *E*_a_ values were much higher within the 100–150 °C range. As can be expected, in this range, the thermal dependence of the viscoelastic functions was more significant on those samples which exhibited higher gel strength.

### 3.3. Lubrication Performance of Oleogels

**Figure 9:**
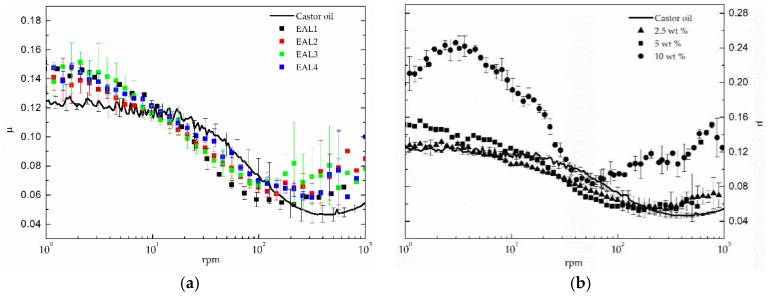
Friction coefficient versus rotational speed curves for epoxidized lignin-based oleogels as a function of (**a**) lignin epoxy index (5 wt % EAL) and (**b**) lignin concentration (EAL1 sample).

The lubrication performance of the epoxidized lignin-based oleogels studied was evaluated in the steel-steel ball-on-three-plates configuration of the tribological cell [24]. Figure 9 shows the friction coefficient versus rotational speed curves obtained under a normal load of 10 N for different tribological systems which include the epoxidized lignin-based oleogels as lubricants. The friction curve obtained using the neat castor oil as a lubricant was also introduced in order to assess the effect of the thickener. The influence of both epoxy index and concentration of the epoxide-modified lignin on the frictional behaviour of these oleogels was analyzed. In general, the evolution of friction coefficient with rotational speed followed the typical behavior described by the Stribeck curve. Initially, starting in the boundary film lubrication regime, the friction coefficient is relatively high and almost constant at low speed. The decrease with the increase of speed in the mixed friction lubrication regime was then detected, prior to an increase once a critical minimum value had been achieved, which preceded the hydrodynamic friction region.

As can be observed in Figure 9a, in the boundary film region, the friction coefficient values for all oleogels are almost constant and are higher than the values found for the base oil, which is probably related to the reduced lubricant entrainment expected in the case of thick oleogels at low rotational speed, when the film thickness is still too narrow [44]. However, oleogels and castor oil friction curves get closer when increasing the rotational speed, finally yielding lower friction for oleogels within the mixed friction lubrication regime. This may be related to the fact that the thickener is able to penetrate into the contact area under this lubrication regime, thus increasing the film thickness and reducing friction. Finally, as expected, higher viscosity of oleogels in comparison with the base oil produces higher values of the friction coefficient along the hydrodynamic lubrication regime, where friction is essentially governed by lubricant properties. On the other hand, the lignin epoxidation degree does not exert a significant influence on the frictional behaviour, although slightly lower values of the friction coefficient were obtained for the oleogel containing the most epoxidized lignin (EAL1) in the mixed friction lubrication regime.

Regarding the thickener concentration, it was found that the oleogel prepared with 10 wt % of epoxide-functionalized lignin (EAL1) produced extremely high values of the friction coefficient within the rotational speed range studied, as a consequence of the highly viscoelastic behaviour. However, oleogels containing the same epoxidized lignin at concentrations lower than 5 wt % generally provide much lower values of the friction coefficient, similar to those provided by the neat castor oil.

## 4. Conclusions

Suitable thickeners for castor oil were obtained by means of chemical modification of alkali lignin (AL) with poly(ethylene glycol) diglycidyl ether (PEGDE) with the aim of developing environmentally friendly lubricating greases. The variation of the input PEGDE/AL ratio in the epoxidation reaction resulted in lignin compounds with different epoxy index and residual NaOH amounts, as a consequence of the two competitive reactions occurring during the synthesis process. Low PEGDE/AL ratios yielded lower consumption of NaOH but favored the etherification of the hydroxyl groups in lignin. Lignins epoxidized at different degrees showed different thermal degradation profiles. The higher the epoxy index, the higher the amount of char, as a consequence of higher etherification of lignin hydroxyl groups.

Epoxidized lignin-based oleogels are highly structured systems, which exhibited the typical viscoelastic response of solid-like gels and the typical yielding flow found in traditional lubricating greases. Moreover, an internal curing process, which promotes the evolution of the rheological properties with time, took place for two months. The values of viscosity, consistency and storage and loss moduli noticeably increased along with both epoxy index and thickener concentration. In addition to this, a power-law increase of the plateau modulus with the epoxy index was found for all thickener concentrations studied, which indicates that a higher epoxy index favors the inter-molecular linkages between lignin and the ricinoleic fatty acid of castor oil. The thermo-rheological characterization of these oleogels revealed a slight thermal dependence of the viscoelastic moduli below 100 °C but a significant softening above this critical temperature. This thermal dependence above 100 °C was more important on those samples with higher crosslinking density. From a tribological point of view, it is worth pointing out that oleogels containing 2.5 and 5 wt % of epoxidized lignin as thickener brought about low friction coefficient values when they were used as lubricants in a steel-steel ball-on-three plates tribocontact, lower than those obtained with the base oil alone within the mixed friction lubrication regime.

## Figures and Tables

**Table 1:** Input PEGDE/AL ratios applied on lignin epoxidation reaction and corresponding epoxy index values and residual NaOH amounts obtained.

Sample	AL (g)	PEGDE (g)	Epoxy index (mol/kg)	NaOH (mol/kg)
EAL1	10	2.5	0.79	5.9
EAL2	10	5	0.52	5.4
EAL3	10	10	0.37	4.7
EAL4	10	20	0.28	2.8
EAL5	10	50	0.15	1.4

**Table 2:** TGA characteristic parameters for the original alkali lignin and epoxidized lignin samples.

Sample	*T*_onset_ (°C)	*T*_max_ (°C)	*T*_final_ (°C)	∆*W* (%)	Residue (%)
AL	130/234/335	150/277/380	221/299/423	5/8/40	47
EAL1	110/241/377	129/277/459	143/304/482	28/7/3	62
EAL2	109/261/375/442	123/277/390/464	144/301/417/489	31/6/2/4	57
EAL3	104/256/324/431	116/285/335/455	135/297/362/483	20/13/11/6	50
EAL4	115/254/322/431	120/274/339/451	142/290/361/475	17/13/15/7	48
EAL5	81/249/337/434	106/305/350/465	112/314/363/487	7/25/30/4	34

**Table 3:** Unworked penetration values and NLGI grade (ASTM D217) for EAL-based oleogels.

EAL Sample	EAL concentration (wt %)	Penetration (mm/10)	NLGI consistency number
EAL1	10	171	4
EAL1	2.5	350	0
EAL1	5	246	3
EAL2	5	316	1
EAL3	5	344	1
EAL4	5	328	1

**Table 4:** Activation energy values for EAL-based oleogels.

EAL sample	*E*_a_ (kJ/mol)	
	5–100 °C	100–150 °C
EAL1	1.2	148
EAL2	0.1	116
EAL3	5.8	98
EAL4	7	96
